# In-plane current induced nonlinear magnetoelectric effects in single crystal films of barium hexaferrite

**DOI:** 10.1038/s41598-022-09363-x

**Published:** 2022-03-30

**Authors:** Maksym Popov, Igor Zavislyak, Hongwei Qu, A. M. Balbashov, M. R. Page, G. Srinivasan

**Affiliations:** 1 Faculty of Radiophysics, Electronics and Computer Systems, Taras Shevchenko National University of Kyiv, Kyiv, 01601 Ukraine; 2 Department of Physics, Oakland University, Rochester, MI 48309 USA; 3 Department of Electrical Engineering and Computer Science, Oakland University, Rochester, MI 48309 USA; 4 National Research University MPEI (Moscow Power Engineering Institute), Moscow, 111250 Russia; 5 Materials and Manufacturing Directorate, Air Force Research Laboratory, Wright-Patterson Air Force Base, Dayton, OH 45433 USA

**Keywords:** Materials science, Physics

## Abstract

This report is on the observation and analysis of nonlinear magnetoelectric effects (NLME) for in-plane currents perpendicularly to the hexagonal axis in single crystals and liquid phase epitaxy grown thin films of barium hexaferrite. Measurements involved tuning of ferromagnetic resonance (FMR) at 56–58 GHz in the multidomain and single domain states in the ferrite by applying a current. Data on the shift in the resonance frequency with input electric power was utilized to estimate the variations in the magnetic parameter that showed a linear dependence on the input electric power. The NLME tensor coefficients were determined form the estimated changes in the magnetization and uniaxial anisotropy field. The estimated NLME coefficients for in-plane currents are shown to be much higher than for currents flowing along the hexagonal axis. Although the frequency shift of FMR was higher for the single domain resonance, the multi-domain configuration is preferable for device applications since it eliminates the need for a large bias magnetic field. Thus, multidomain resonance with current in the basal plane is favorable for use in electrically tunable miniature, ferrite microwave signal processing devices requiring low operating power.

## Introduction

Various signal-processing devices such as filters, phase shifters, and isolators are widely used in radars and communication systems at microwave and millimeter-wave bands^1^. Such devices allow control over microwave signal properties (magnitude, phase, polarization, frequency spectrum etc.). Microwave components with reconfigurable characteristics, in particular, are highly sought after since such property can greatly increase the functionality of the devices^2–4^. In this regard ferrite devices have desirable properties such as low losses, but traditional tuning of the devices is achieved with an external bias magnetic field^5,6^. The drawback of this approach is the need for a source of variable bias magnetic field in the form of electromagnets or solenoids which increases device dimensions, weight and electric power requirements. In their search for alternative ways to tune high frequency magnetic devices researchers have focused their attention on purely electrical tuning^7–12^. Tuning of magnetic parameters by applying an electric field was demonstrated in magnetic semiconductors^7^, in magnetic materials by spin polarized electric current (spin-transfer-torque effect)^8^, and via spin–orbit torque^9^. The most promising technique so far has been electric field tuning by piezoelectric strain induced in a composite of ferrite and ferroelectric layers^10–12^.

We reported recently on a novel current-induced nonlinear magnetoelectric (NLME) effect in hexagonal ferrites that has the potential for a new family of electric field/current tunable ferrite devices^13–16^.This effect was first observed in M-type strontium (Sr) and barium (Ba) hexagonal ferrite thick films under a current along the hexagonal c-axis^13–15^. The effect manifests itself as modification of the saturation magnetization and magnetocrystalline anisotropy field. The origin of NLME is likely weakening of the super exchange interaction due to exclusion of the electrons carrying the current from contributing to magnetic interactions^13^. Ferromagnetic resonance (FMR) measurements were done as a function of the input electric power *P* to obtain data on shift in FMR and was used to estimate the changes in magnetization and anisotropy field that were found to be directly proportional to *P* (or square of the electric field *E*). The NLME, however, has been investigated so far only in the specific experimental configuration when the current flows along the symmetry axis (*c*-axis) of the highly anisotropic M-type hexagonal ferrites (barium and strontium hexaferrites)^13–15^ or along the easy plane in the case of Y-type ferrites^16^.

Here we report on the observation of a strong NLME interaction in M-type hexagonal ferrite for currents in the basal plane, perpendicular to the c-axis. Such an investigation is of fundamental and technological importance, in particular, for dual electric and magnetic field tunable high frequency devices. Theoretical predictions on NLME due to basal plane currents suggest that this effect should be strong^14,17^. A thick film of BaFe_12_O_19_ (BaM) obtained from a single crystal platelet and thin films grown by liquid phase epitaxy (LPE) were used in this study. Ferromagnetic resonance measurements were done in the frequency range 55–58 GHz in the multidomain and single domain states of the film with a current in the c-plane. The shift in FMR frequency was found to increase linearly with input electric power and was larger in the single domain state than for the multidomain resonance. The variations in the magnetic parameters and NLME tensor coefficients have been estimated from these data and compared with the previously reported case of the current along the hexagonal c-axis. Results of the study indicate strong NLME interactions for the in-plane currents and the potential for electric field tunable miniature M-type hexaferrite planar devices in the millimeter wave band.

## Experimental set-up and measurements

**Figure 1:**
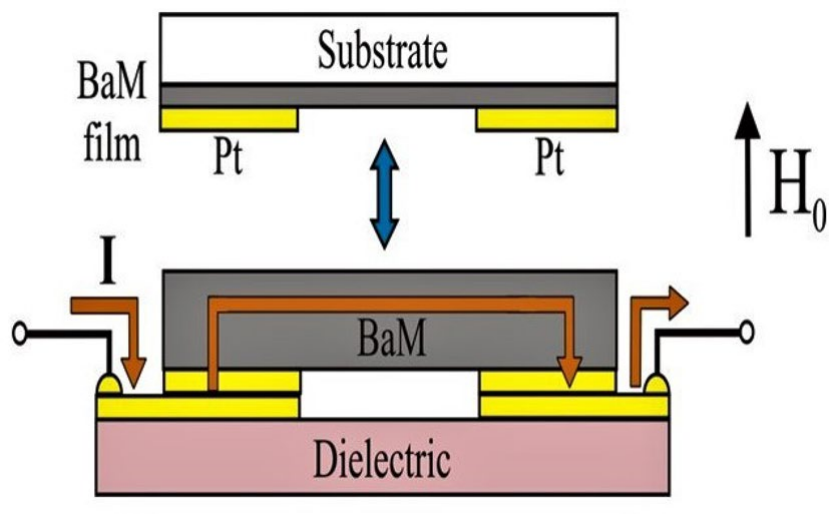
Schematic diagram showing the experimental setup for the investigation of the nonlinear magnetoelectric (NLME) effects in barium hexaferrite for the in-plane currents.

Two types of barium ferrite samples were used in this study. A single crystal platelet of barium hexagonal ferrite, Ba Fe_12_O_19_, grown by floating zone techniques (provided by Professor Balbashov, Moscow Power Engineering Institute, growth details in Ref.^18^) and a thin-film sample grown by liquid phase epitaxy (LPE) on a non-magnetic substrate were used. The single crystal platelet was cut into a 2.5 mm × 2.0 mm rectangle with *c*-axis perpendicular to the plane and polished down to 150 μm in thickness. The LPE grown film, 4 μm in thickness was cut to lateral dimensions 1.0 mm × 2.1 mm. Two platinum electrodes separated by ~ 1 mm were deposited on the sample plane to provide the electrical contacts for passing a current as shown on the Fig. 1. The measured small-signal electrical resistance was *R*_*b*_ = 12.7 kΩ for bulk sample and *R*_*f*_ = 360 kΩ for the LPE film.

The samples with the electrodes were bonded to the supporting fixture made from low-loss microwave dielectric (see Fig. 1) and positioned inside a U-band rectangular waveguide. Then the transmission characteristics of the sample were studied using a vector network analyzer (Agilent Tech., E8361A) and measuring the scattering matrix parameter *S*_21_ as a function of frequency *f* for a series of external bias magnetic field values. Measurements were made with and without applied electric current. For the case of BaM thin film the absorption in the transmission mode was too small for reliable measurements. Therefore, the sample was positioned at the open flange at the cross-section of the waveguide. Then profiles of the reflection coefficient *S*_*11*_ was recorded as the frequency was swept. The magnetic field in all the cases was applied along the hexagonal axis (i.e. perpendicularly to the basal plane).

## Results

**Figure 2:**
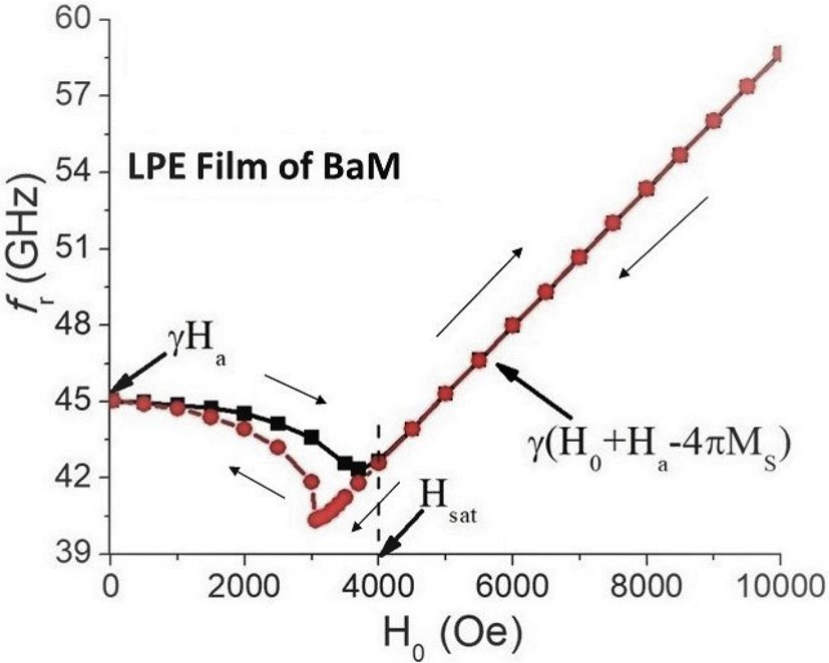
The resonance frequency *f*_*r*_ as a function of bias magnetic field *H*_*o*_ for the FMR in LPE thin-film barium hexaferrite sample.

The FMR resonance frequency *f*_*r*_ was determined from the minimum of transmission/reflection characteristics as a function of the bias field *H*_0_. Data on the dependence of *f*_*r*_ on *H*_0_ is shown in Fig. 2 for the LPE thin-film sample (similar data for the thick film of BaM are shown in Fig. S1 in the supplement). One can see two different regions on this plot: (i) multi-domain state with rather weak dependence of *f*_*r*_ on *H*_0_ and (ii) saturated state with linear relation between the resonance frequency and bias magnetic field. The magnetic field *H*_*sat*_ at which the transition to saturated state takes place was defined as a point of divergence between the curves showing variation of *f*_*r*_ with *H*_0_ measured for increasing and decreasing bias fields. These data were then used to extract initial magnetic parameters of the sample. The expressions for the FMR frequency in the unsaturated (multi-domain) and saturated (single-domain) states, respectively, are given by^13^:

1 $$f_{r} \left( {H_{0} = 0} \right) = \upgamma H_{a} ,$$

2 $$f_{r} \left( {H_{0} } \right) = \upgamma (H_{0} + H_{a} - N_{zz} {4}\pi M_{S} )$$

In the expressions above, *γ* is the gyromagnetic ratio, *H*_*a*_ is the uniaxial anisotropy field, *M*_*S*_ is the saturation magnetization, and *N*_*zz*_ is an equivalent demagnetizing factor of the sample. The estimated *N*_*zz*_ values for the thin film sample was *N*_*zz*_ ≈ 1 and for bulk thick film *N*_*zz*_ ≈ 0.85^19^.

Equation (2) is approximate since it describes spatially uniform magnetization oscillations which may not exist in a rectangular sample. For a flat ferrite sample with thickness much less than the lateral dimensions, it may be regarded as a good approximation.

The magnetic parameters for the samples were evaluated from FMR data on resonance frequency using the following procedure. First, the resonance frequency for the single domain state *f*_*r*_ was measured as a function of *H*_*0*_ and the data was fitted to Eq. (2) to obtain the gyromagnetic ratio *γ* and (*H*_*a*_–*N*_*zz*_4*πM*_*S*_*).* Then, from the zero-field mode frequency and Eq. (1) the uniaxial anisotropy field *H*_*a*_ was determined. Finally, with known *H*_*a*_ and *N*_*zz*_ the saturation magnetization 4*πM*_*S*_ was calculated. The values of these parameters for the single crystal thick film are *γ* = 2.75 GHz/kOe, 4*πM*_*S*_ = 4.95 kG and *H*_*a*_ = 17 kOe. The corresponding values for the LPE thin film are 2.67 GHz/kOe, 4.9 kG and 16.9 kOe, respectively.

**Figure 3:**
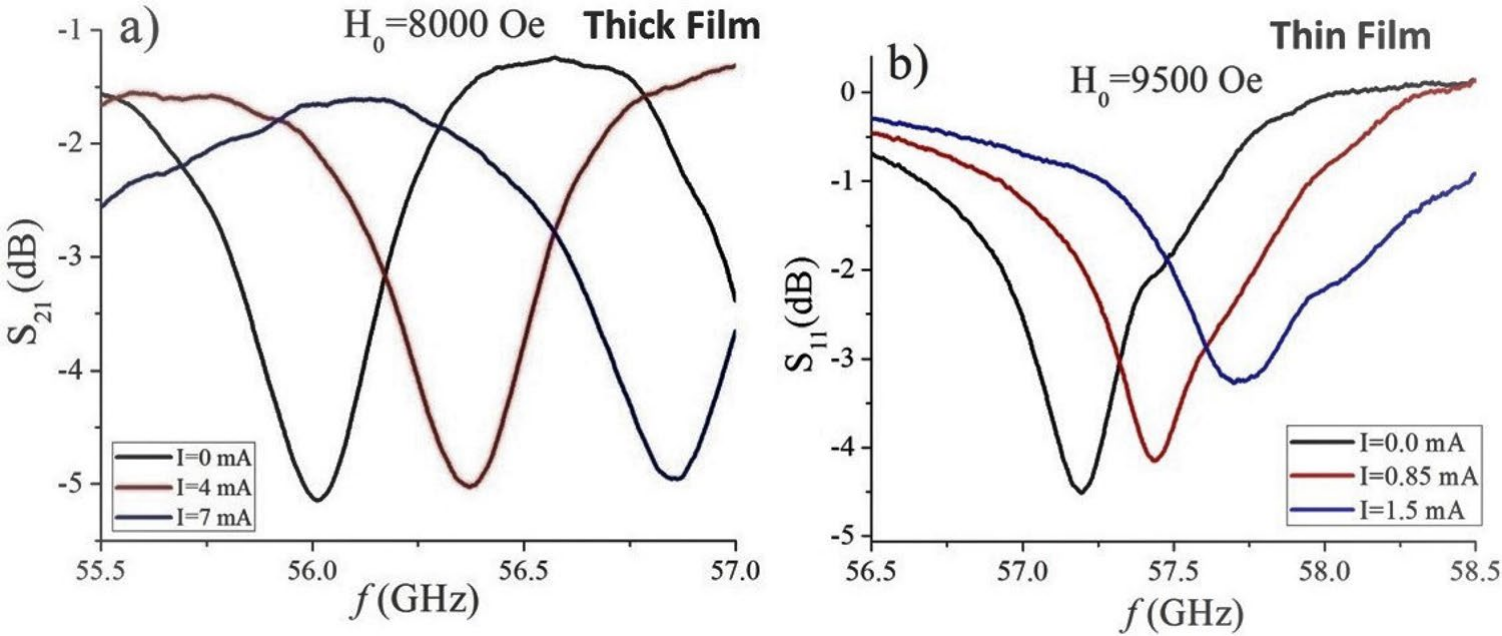
(**a**) Profiles of transmission coefficient as a function of *f* showing FMR in BaFe_12_O_19_ single crystal platelet and (**b**) data on variation of reflection coefficient with. *f* showing FMR in LPE film of BaM. The data are for single domain resonance for a series of pulsed DC electric currents.

For studies on the NLME effects FMR measurements under single domain and multidomain states were carried out as a function of the direct current (DC) current through the sample for a constant bias magnetic field. Representative transmission/reflection characteristics for FMR under single domain state for fixed *H*_*o*_ and a series of DC current in the basal plane are shown in Fig. 3.

The DC current was supplied in short pulses (≈ 1 s) in order to avoid Joule heating. The data in Fig. 3 indicate that the tuning was achieved for a relatively small DC current, < 2 mA for the LPE film and for < 10 mA for the thick film and for applied voltages of 0–160 V for the LPE film and 0–40 V for the thick platelet.

**Figure 4:**
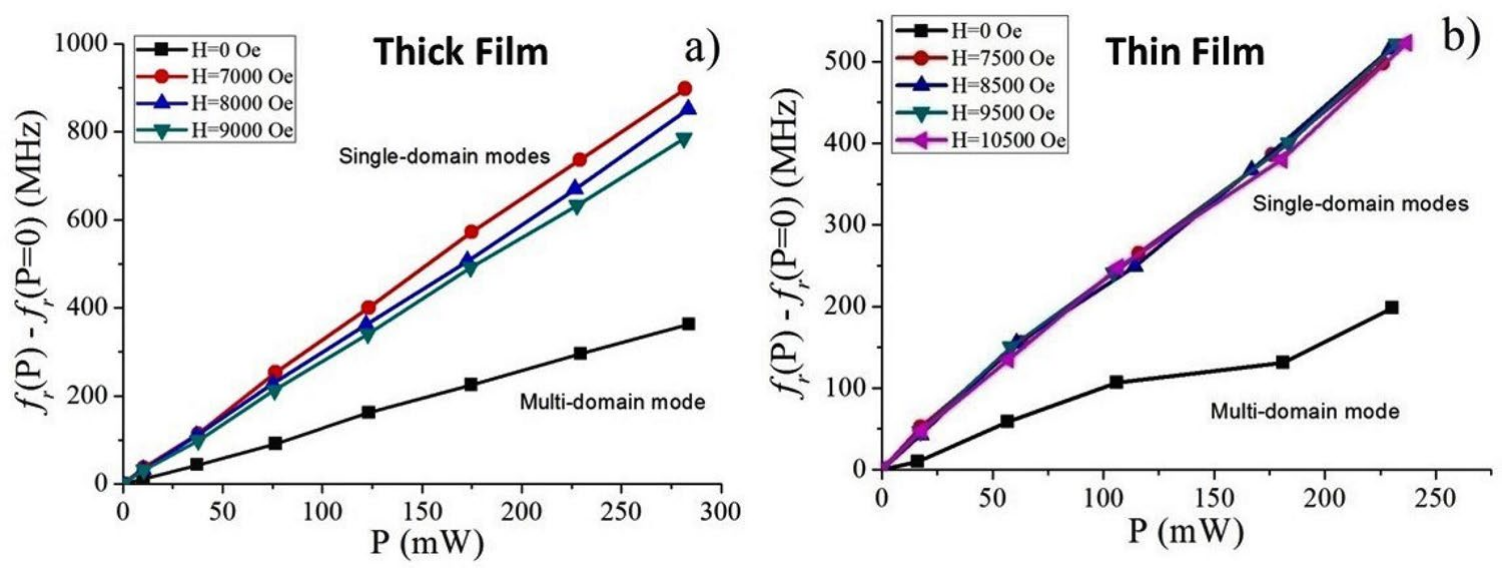
Frequency shift for FMR in the multi-domain and single-domain states as a function of applied DC power for (**a**) thick film and (**b**) thin-film of barium hexaferrite for the current flowing in the basal plane.

Figure S2 in the supplement shows data on *S*_*21*_ as a function of *f* for FMR in BaFe_12_O_19_ single crystal thin film and variation in *S*_*11*_ with *f* for thick film of BaM for multi-domain resonance for a series of pulsed DC electric currents. From the single domain FMR profiles (as in Fig. 3 and in Figs. S3 and S4 in the supplement) and multidomain FMR measurements (Fig. S2 in the supplement) we obtained data on the dependencies of the shift in the FMR frequencies as a function of the input electric power *P* = *UI* that are shown in Fig. 4. The data are for the thick single crystal film and thin LPE film for the multi-domain case (in the absence of the external bias) and in the magnetically saturated or single-domain state. The data show a shift in the FMR frequency that is linearly proportional to the applied power, a similar behavior as for the current along the hexagonal c-axis^11,13^ and in agreement with the theoretical predictions^13,14^.

However, the frequency shift and the slope of frequency variation with power differ for the two films and in the single domain and multidomain states. For the same input power, the frequency shift for the thick film is a factor of two higher than for the thin film. The rate of increase in the frequency shift with the input power in the saturated state is 2 to 3 times higher than for multi-domain state. Also, in the saturated state the frequency shift shows only a weak dependence on the bias magnetic field.

**Figure 5:**
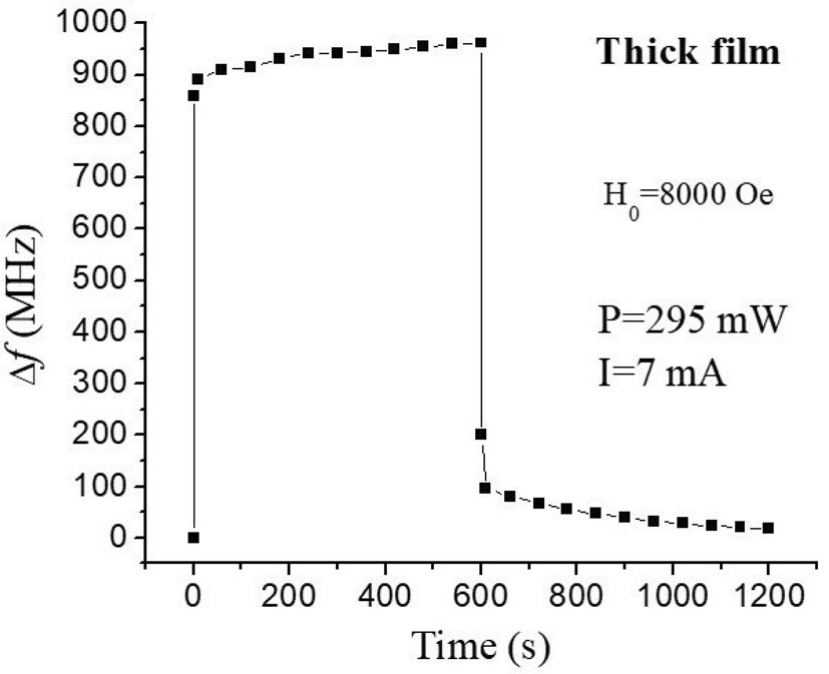
FMR frequency shift as a function of time when a DC current was passed through the thick film of BaM. The frequency shift was measured when the current was on for 10 min and then when it was turned off.

Finally, instead of pulsed DC currents the samples were subjected to continuous DC currents in order to determine the contribution from the Joule heating to the observed frequency shifts in Fig. 4. Indeed, the rise of sample’s temperature should lead to a decrease in the saturation magnetization and an increase in the FMR frequency, thus mimicking the consequences of the NLME effect. Figure 5 shows the influence of Joule heating on the measured frequency shift for the thick film of BaM. A current of 7 mA corresponding to input power of 295 mW was passed through the sample for 10 min and then switched off. The frequency shift was measured for single domain resonance as a function of time with the current on and then when it was turned off.

Results in Fig. 5 show that for the thick films the continuous application of current for 10 min duration led to a change in *f*_*r*_ that amounted to 85 MHz for the applied DC power of 294 mW. This frequency shift is an order of magnitude smaller than the 860 MHz shift due to the NLME effects. Yet one must stress that results presented here were for pulsed currents for a short duration of 1 s and, therefore, the actual effect of sample heating is well below the value for continuous currents. Nevertheless, this result agrees with our previous evaluations of Joule heating by various techniques, including direct measurements^11–13^ which showed that Joule heating accounts for no more than 10% of the observed FMR frequency variation. For the LPE film the influence of heating was more pronounced due to much smaller volume of the ferrite material. Thus, even for the 150 mW of applied DC electric power the frequency shift after 10 min duration was105 MHz. These data demonstrate that thin films have a large heating related effects in comparison to thick films.

## Analysis and discussion

**Figure 6:**
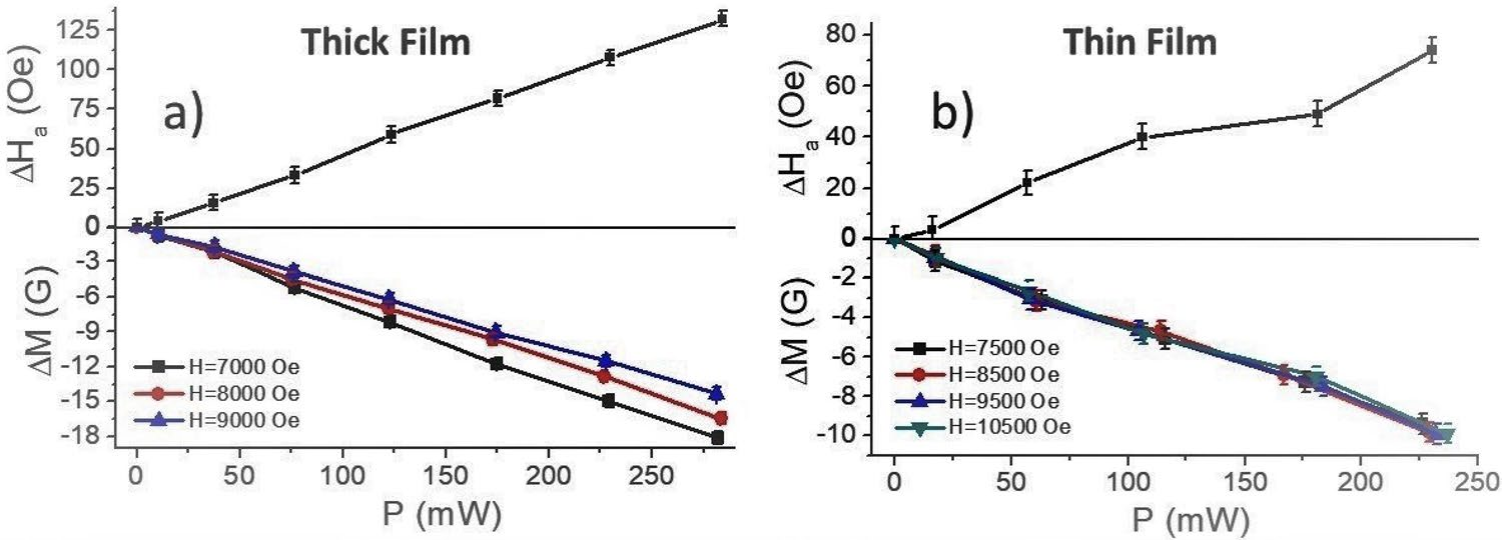
Variations in the saturation magnetization and the uniaxial anisotropy field due to NLME in (**a**) thick film and (**b**) thin film of barium ferrite.

The results in Fig. 4 were used to estimate the variation in the magnetic parameters and the NLME coefficients as described in next. The NLME induced variations in the anisotropy field ΔHa and the magnetization ΔM were calculated from the shift in the FMR frequency in the multidomain and single domain states. The procedure used was the same as the one used for estimation of the magnetic parameters from the FMR data in Fig. 2. The change in the anisotropy field was estimated from data on shift in the multidomain resonance frequency as a function of current and then the change in the magnetization from variation in effective magnetization calculated from single domain resonance frequencies. Figure 6 shows estimated variations in the anisotropy field ΔH_a_ and the saturation magnetization ΔM as a function of input electric power for both the thick and thin films of BaM. A linear increase in ΔH_a_ and a decrease in ΔM are observed for both films.

The results in Fig. 6 were used to estimate the NLME coefficients based on the phenomenological theory developed in Refs.^13,14^. More precisely, the theory predicts that the application of electrical current in the basal plane of M-type hexaferrite leads to the following changes in saturation magnetization *M*_*s*_ and uniaxial anisotropy energy constant *K*_*u*_

3 $$\Delta {M}_{S}(p)=p{\gamma }_{311}{\rho }_{\perp }$$

4 $$\Delta {K}_{u}(p)\approx p\left({\delta }_{3311}-\frac{{\delta }_{1111}+{\delta }_{1122}}{2}\right){\rho }_{\perp }$$

where *p* = *P/V* is the electric power volume density, $\rho_ \perp$ is the resistivity of the sample in the direction perpendicular to hexagonal axis, and *γ*_ijk_ and *δ*_ijkl_ are the third- and fourth-order NLME tensor coefficients which are determined by crystalline and magnetic symmetry of the ferrimagnet^20–22^. In Eq. (3) the first index in *γ*_ijk_ refers to the direction of the magnetization vector (which is *z* or *3*) whereas the second and third indices denote the bilinear combination of applied electric field components *E*_*j*_*E*_*k*_. Since in our model the basal plane is isotropic, we may, without losing generality, attribute index *1* to the direction of the in-plane electric field. This leaves only one non-zero term, namely *E*_1_*E*_1_ = *E*^*2*^*,* and, the term γ_*311*_ in Eq. (3). In the case of *δ*_ijkl_ the first pair of indices denote the bilinear combination of magnetization components *M*_*i*_*M*_*j*_ and the second pair denote the electric fields.

The uniaxial anisotropy field *H*_*a*_ in Eqs. (1) and (2) is given by *H*_*a*_ = 2*K*_*u*_/*M*_*S*_, and for small variations in the magnetic parameters, Δ*K*_*u*_ <  < *K*_*u*_ and *ΔM*_*S*_ <  < *M*_*S,*_* ΔH*_*a*_ is also a linear function of the power density *p* and is given by

5 $$\Delta {H}_{a}(p)\approx {H}_{a}(p=0) \cdot \left(\frac{{\delta }_{3311}-\frac{{\delta }_{1111}+{\delta }_{1122}}{2}}{{K}_{u}}-\frac{{\gamma }_{311}}{{M}_{S}}\right){\rho }_{\perp }p$$

Equations (1) and (2) together with Eqs. (3) and (5) account for the measured variations in the magnetic parameters. Indeed, from Eqs. (1) and (5) it follows that due to NLME the zero-field FMR mode should experience a frequency shift Δ*f*_*r*_ (*H*_0_ = 0) = γΔ*H*_*a*_ that is linearly proportional to the power density. On the other hand, in the saturated state for fixed bias fields the shift is defined by changes in both *H*_*a*_ and *M*_*s*_: Δ*f*_*r*_ (*H*_0_) ≈ γ(Δ*H*_*a*_–*N*_*zz*_4*π*Δ*M*_*S*_) and, therefore, for the situation when *ΔM*_*s*_(*p*) < 0 (i.e., *γ*_311_ < 0) the frequency change will always be larger than in the case of *H*_*0*_ = 0.

The average FMR frequency tuning efficiency measured for the thick film sample ∂*f*_*r*_*/∂P* = 2.99 ± 0.20 MHz/mW for the single domain and (1.29 ± 0.01) MHz/mW for the multidomain resonances, respectively. The corresponding tuning efficiencies for the thin film sample are ∂*f*_*r*_*/∂P* = 2.17 ± 0.05 MHz/mW and 0.81 ± 0.07 MHz/mW. These tuning efficiency values are of an order of magnitude higher than for currents along the hexagonal c-axis in SrM and SrAlM^13,14^, although for the thick film sample the tuning efficiency is somewhat larger. Thus, the use of saturated state for frequency agile ferrite microwave devices is preferable since it provides higher tuning efficiency. However, it should be noted that a source of external bias magnetic field is required to saturate the sample. Therefore, the devices using this state will be bulkier and heavier. On the other hand, device utilizing multi-domain state does not need any external magnetic system and thus may be potentially miniature in size, lightweight and planar (with the use of stripline or coplanar line instead of waveguide). In such cases the frequency tuning required for tunable device operation will be provided by purely electrical means.

Parameters for electric field control of the magnetic parameters due to NLME were extracted from the data on *Δf*_*r*_ (*P*) dependence on input DC power in Fig. 4 and using Eqs. (1) and (2) and their values are given in Table 1. The application of electric field results in decrease of both the saturation magnetization and uniaxial anisotropy energy constant. However, since the magnetization *M*_*S*_ decreases at a faster rate than *K*_*u*_, the anisotropy field *H*_*a*_ = 2*K*_*u*_/*M*_*S*_, increases with the input power. According to Eq. (3) ∂*M*_*S*_*(P)/∂P* = *γ*_311_ ${\rho }_{\perp }$/*V* and thus the corresponding third order.

**Table 1:** Magnetic and magnetoelectric parameters of single crystal thick film and LPE thin film of M-type barium hexaferrites.

Magnetic or magnetoelectric parameter	BaFe_12_O_19_ (film)	BaFe_12_O_19_ (bulk)
γ, MHz/Oe	2.67	2.75
4*πM*_S_, G	4900	4950
*H*_*a*_, kOe	16.9	17.0
*K*_*u*_, erg/cm^3^	3.29·10^6^	3.35·10^6^
Specific resistance ρ_⊥_, Ω mm	1600	2800
∂*f*_*r*_*/∂P*, MHz/mW	0.81 (*H*_0_ = 0) 2.17 (*H*_0_ > *H*_sat_)	1.29 (*H*_0_ = 0) 2.99 (*H*_0_ > *H*_sat_)
∂*H*_*a*_ /∂*P,*Oe/mW	0.30	0.47
∂*M*_S_/∂*P*, G/mW	– 0.04	– 0.06
*∂K*_*u*_*/∂P*, erg/(cm^3^ mW)	– 285	– 401
|γ_311_|_,_ G mm^2^/(W Ω)	90·10^–6^	8600·10^–6^
$\left|{\delta }_{3311}-\frac{{\delta }_{1111}+{\delta }_{1122}}{2}\right|$, erg/(W Ω mm)	0.6·10^–3^	58·10^–3^

NLME coefficient may be determined from its value in Table 1. Similarly, a linear combination of fourth order NLME coefficients was estimated from Eq. (4). The experimental technique utilized here did not allow one to extract the values of each one of the coefficients δ_1111_, δ_3311_, and δ_1122_.

From the estimated values of *γ*_311_ it is clear that the largest value of this ME coefficient is measured for the single crystal platelet and is equal to 8600·10^−6^G mm^2^/(W Ω), whereas it is much smaller for the LPE film. The higher coupling coefficient for the bulk single crystal platelet than for the thin LPE film may be accounted for by the larger current in the bulk sample than for the LPE film. We proposed a phenomenological model for NLME effects in Ref. ^13^. According to this model the likely origin of the ME effect is the conduction current in the hexagonal ferrite due to the presence of divalent Fe and hopping of electrons between divalent and trivalent Fe. It was suggested that the Fe ions involved in the conduction process are excluded from participating in the super-exchange interactions and spin orbit interactions, resulting in changes in the magnetization and anisotropy field. Thus, one would expect the coupling to be strong in samples that carry a large current. This accounts for the higher ME coupling coefficient for bulk sample with R = 12.7 kΩ than for the LPE film with R = 360 kΩ.

We can compare these ME coupling coefficient values for the samples studied here with the earlier published results on materials with the same or similar chemical composition for the current flowing along the *c*-axis. In that case only the *E*_3_ component of electric field is non-zero and thus the only relevant NLME tensor coefficient is *γ*_333_ (coefficient for static magnetic fields and electric fields all along the z-axis or direction *3*). Specifically, the |*γ*_333_| value for pure barium hexaferrite evaluated from data presented in Ref.^15^ was 45·10^−6^G mm^2^/(W Ω) and for the *Al*-substituted strontium hexaferrites (SrAl_x_Fe_12-x_O_19_) was |*γ*_333_| = 317·10^−6^G mm^2^/(W Ω)^13^. Thus, the NLME coefficients for the current in basal plane in thick film of BaM is at least an order-of-magnitude higher than for the current along the *c*-axis for M- type ferrites. In the case of LPE film of BaM *γ*_311_, although smaller, is still higher than previously reported *γ*_333_ values for fields along the c-axis for BaM. The origin of this enhancement in the strength of the ME coupling interactions in the case of in-plane currents than for the current along the c-axis needs to be investigated. One therefore needs a first principles model for the NLME effects for such an understanding of these observations.

Finally, we compare the tuning efficiency for the films studied here with results for single crystal platelets of Zn_2_Y which is a hexagonal ferrite with easy plane anisotropy. An NLME induced frequency shift of 1200 MHz to 1500 MHz was measured in Zn_2_Y for input power of 90 mW for this case and is higher than for the BaM films studied here^16^. One may compare the tuning efficiency measured for in-plane currents in BaM with tuning of FMR in ferromagnetic-piezoelectric composites in which the tuning results from mechanical strain in the piezoelectric phase due to an applied DC voltage^12^. In the case of composites, the input DC power is negligible due to very low current and although the tuning mechanisms are different for the ME effects in the composites and NLME effects in the hexaferrites, a comparison could be made in terms of rate of change in *f*_*r*_ with voltage *V*. Studies on voltage tuning of FMR were reported on several ferrimagnet/ferromagnet-ferroelectric composites^23–32^. Early works on yttrium iron garnet (YIG)-PZT reported *Δf*_*r*_*/ΔV* = 0.04 MHz/V and the low tuning rate is due to low magnetostriction for YIG^23,25^. A similar low tuning rate was reported for composites of SrM-PZT^26^. In composites with nicklel ferrite (NFO) films on ferroelectrics, FMR could be tuned by 0.25 MHz/V^24^. For Fe_3_O_4_ films on ferroelectrics *Δf*_*r*_*/ΔV* amounted to 0.75 MHz/V and a much higher rate of 70 MHz/V was measured in a coaxial nanofiber of NFO and PZT^30^. Ferromagnetic alloys with PZT also showed a large tunability of FMR with the application of a DC voltage across the ferroelectric layer^29^. In this study we measured maximum value of *Δf*_*r*_*/ΔV* = *3 MHz/V* for thin films and 24 MHz/V for the thick film.

## Conclusions

We reported here on electric field/current-induced NLME effect in barium hexaferrite. Single crystal platelets and thin films made by LPE were used for studies on NLME under the current in the crystallographic basal plane. The M-type hexaferrites is of interest for the use in millimeter-wave band signal-processing devices due to the presence of a very large internal anisotropy field. Another key advantage for use of the ferrites in the high frequency devices is the presence of a zero-field multidomain resonance mode. For both thick and thin films it was shown that the in-plane current resulted in up shift of the FMR frequency which was linearly proportional to the applied DC electric power, in agreement with previously developed theoretical models^12–14^. Measurements on NLME were carried out for two different types of FMR modes: (i) multi-domain resonance under which the frequency tuning due to NLME is relatively low but is achieved in the absence of external bias magnetic field (for *H*_0_ = 0) making this configuration preferable for the practical devices since the need for an external bias magnetic field is eliminated; and (ii) resonance under single domain state under which a much larger frequency shift due to NLME was measured (which is also nearly independent of the bias magnetic field).

The NLME coefficients were determined from the shift in the resonance frequency and the estimated variations in the magnetic parameters. The third order NLME tensor coefficient *γ*_311_value was evaluated for the single crystal platelet and was found to be much higher than the *γ*_333_ value that corresponds to the current flow along the hexagonal axis for M-type hexaferrites. The *γ*_311_ value for the LPE thin film was smaller than for the thick film, but still higher than *γ*_333_ for the single crystal barium hexaferrite. The influence of Joule heating was found negligible for the short DC pulses used in these investigations of NLME effects.

## Supplementary Information

Supplementary Figures.
