# Supplementary material for: In-plane current induced nonlinear magnetoelectric effects in single crystal films of barium hexaferrite
Source: Sci Rep. 2022 Mar 30;12:5374. doi: 10.1038/s41598-022-09363-x (PMC8967819; doi:10.1038/s41598-022-09363-x)

Supplementay Information

In-plane Current Induced Nonlinear Magnetoelectric Effects in Single Crystal Films of Barium Hexaferrite

Maksym Popov,[1,2] Igor Zavislyak,[1] Hongwei Qu,[3] A. M. Balbashov,[4] M.R. Page,[5] and G. Srinivasan[2*]

[1] *Faculty of Radiophysics, Electronics and Computer Systems, Taras Shevchenko National University of Kyiv, Kyiv, 01601, Ukraine*

[2] *Department of Physics, Oakland University, Rochester, MI 48309, USA*

[3] *Department of Electrical Engineering and Computer Science, Oakland University, Rochester, MI 48309, USA*

[4] *National Research University MPEI (Moscow Power Engineering Institute), Moscow, 111250, Russia*

[5] *Materials and Manufacturing Directorate, Air Force Research Laboratory, Wright-Patterson Air Force Base, Dayton, Ohio 45433, USA*

*Corresponding author: Gopalan Srinivasan

E-mail address: srinivas@oakland.edu

Figure S1: The frequency vs. field dependence for the FMR in single crystal thick film of barium hexaferrite sample.

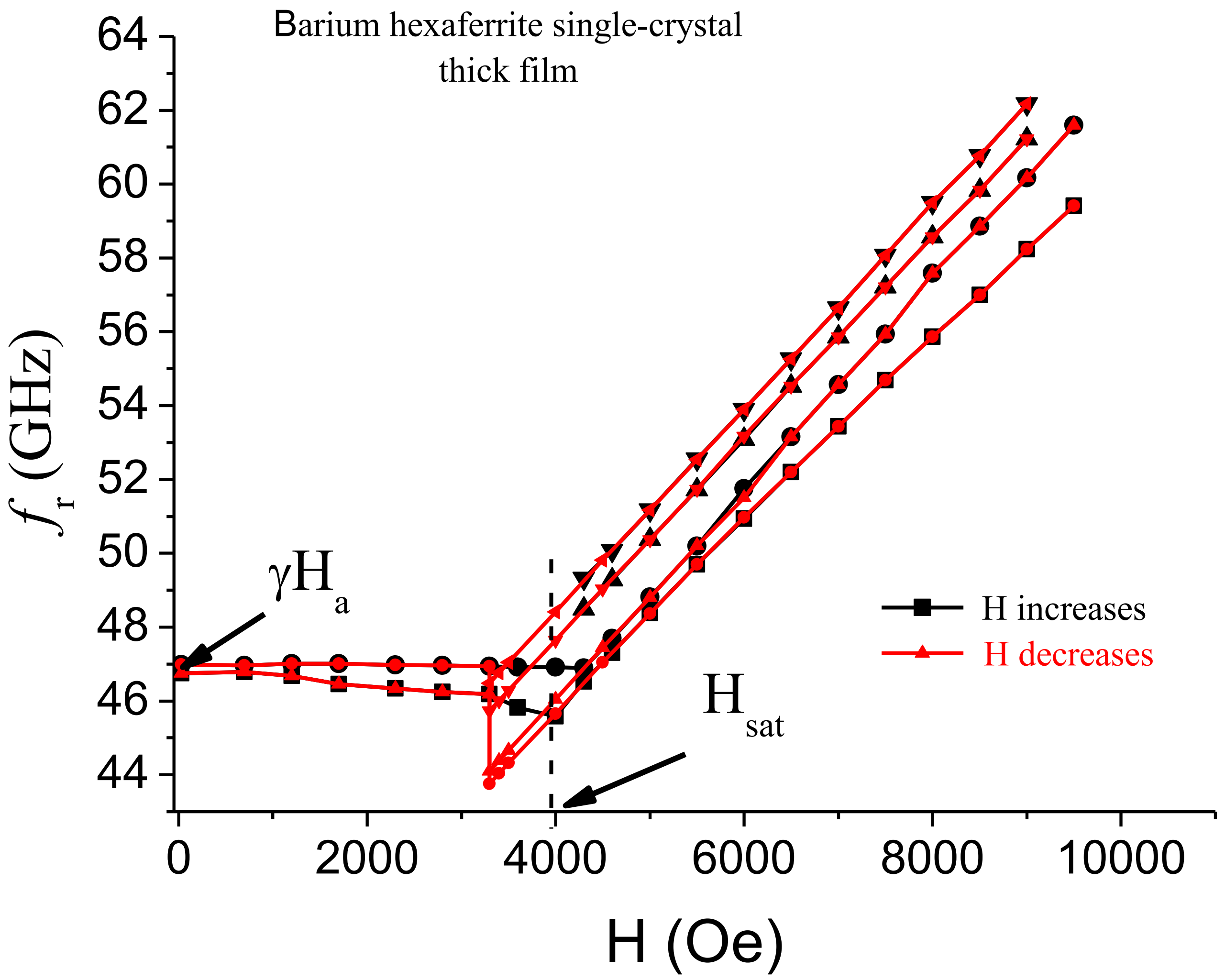

Figure S2 - (a) Transmission coefficient vs. *f* data showing FMR in $BaFe_{12}O_{19}$ single crystal thin film and (b) reflection coefficient vs. *f* data for thick film of BaM. The data are for multi-domain resonance for a series of pulsed DC electric currents.

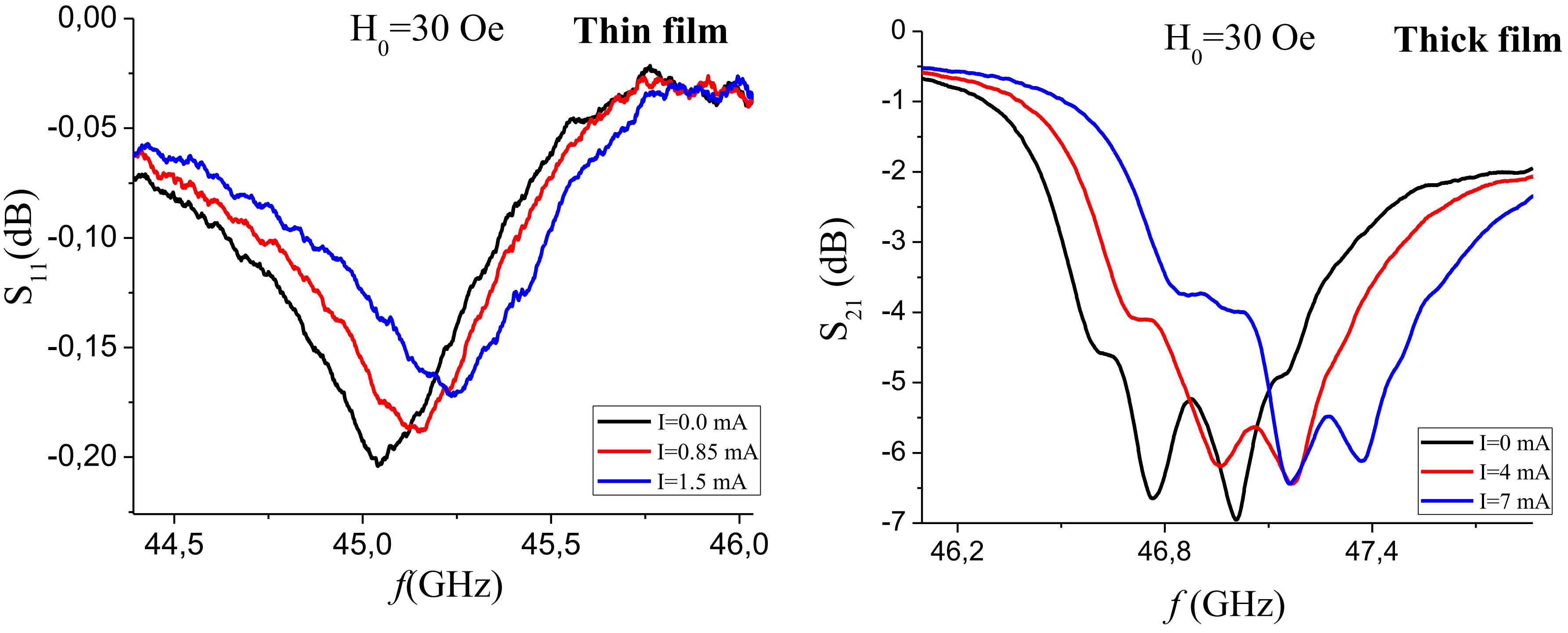

Figure S3- Resonance profiles as a function of current for single domain resonance for Ho = 7500 and 8500 Oe for thin film of BaM).

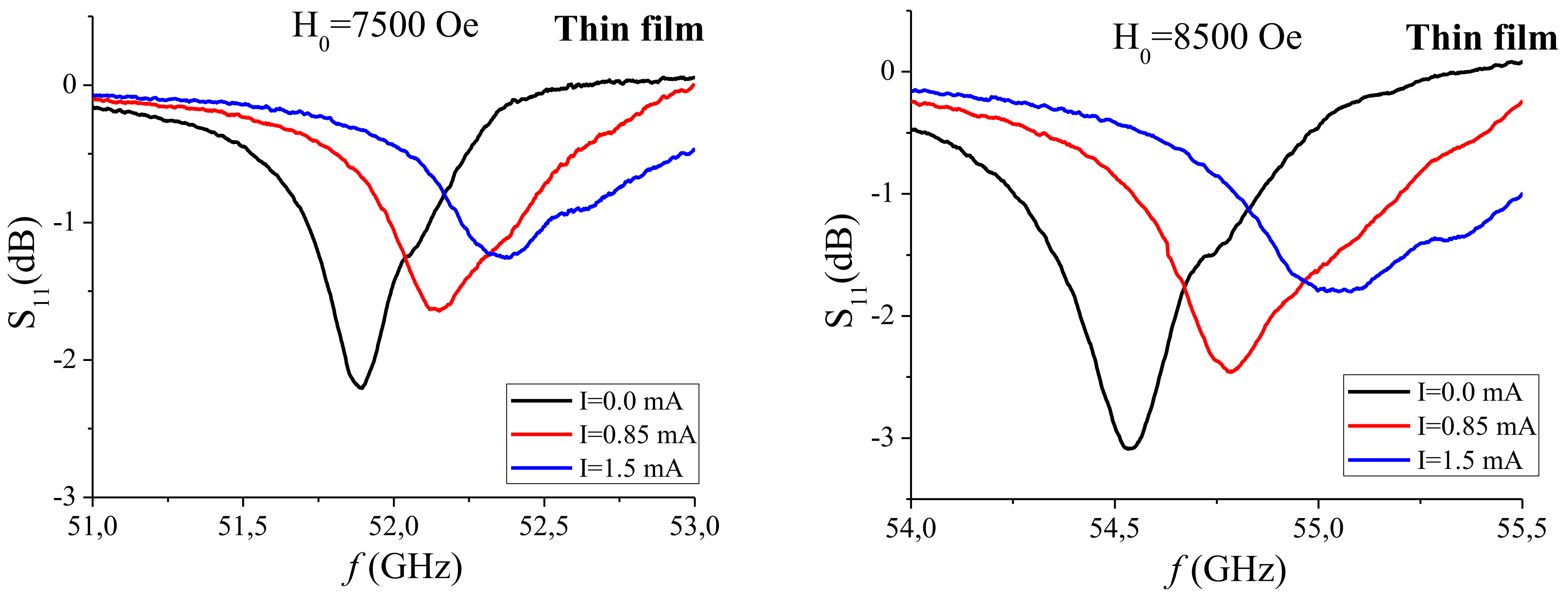

Figure S4- Resonance profiles as a function of current for single domain resonance for Ho = 7000 and 9000 Oe for thick film.

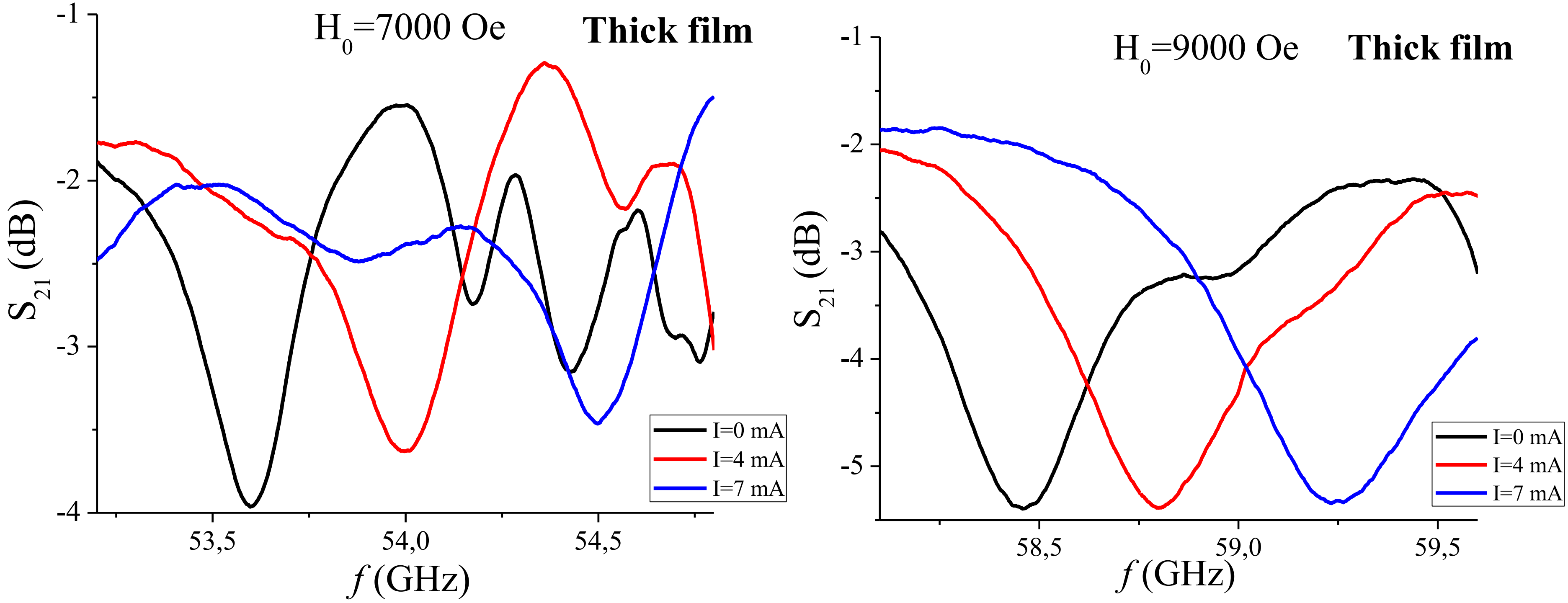

Supplement: Supplementary file 1 — Supplementary Figures. [file 41598_2022_9363_MOESM1_ESM.pdf]
